# Praxis der medikamentösen Thromboseprophylaxe und Antikoagulation bei Patienten mit Sepsis und vorbestehender Antikoagulation oder Heparin-induzierter Thrombozytopenie Typ II – Ergebnisse einer deutschlandweiten Umfrage auf Intensivstationen

**DOI:** 10.1007/s00101-021-01011-9

**Published:** 2021-08-05

**Authors:** Thomas Schmoch, Thorsten Brenner, Andrea Becker-Pennrich, Ludwig Christian Hinske, Markus A. Weigand, Josef Briegel, Patrick Möhnle, SepNet Study Group

**Affiliations:** 1 Klinik für Anästhesiologie, Universitätsklinikum Heidelberg, Heidelberg, Deutschland; 2 Klinik für Anästhesiologie und Intensivmedizin, Universitätsklinikum Essen, Hufelandstr. 55, 45147 Essen, Deutschland; 3 Klinik für Anästhesiologie und Abteilung für Transfusionsmedizin, Zelltherapeutika und Hämostaseologie (ATMZH), LMU Klinikum München, München, Deutschland; 4 Institut für medizinische Informationsverarbeitung, Biometrie und Epidemiologie, LMU München, München, Deutschland

**Keywords:** Septischer Schock, Niedermolekulares Heparin, Vorhofflimmern, Intensivmedizin, Thromboembolien, Septic shock, Low molecular weight heparin, Atrial fibrillation, Intensive care medicine, Thromboembolic complications

## Abstract

**Hintergrund:**

Sowohl eine vorbestehende Antikoagulation als auch prädisponierende Vorerkrankungen für Thromboembolien stellen ein häufiges Problem bei Patienten mit Sepsis dar, wenngleich der Umgang mit diesen Rahmenbedingungen von den aktuellen nationalen und internationalen Leitlinien für Sepsis und septischen Schock nicht adressiert wird. Ein Ziel der hier vorliegenden deutschlandweiten Umfrage war es, den Umgang von Intensivmedizinern mit derartigen Problemen zu eruieren.

**Methoden:**

Von Oktober 2019 bis Mai 2020 führten wir eine deutschlandweite Umfrage unter ärztlichen Leitern von Intensivstationen zum Thema Antikoagulation und medikamentöser Thromboseprophylaxe bei Sepsis und Sepsis-induzierter Koagulopathie durch. Ein Fokus war hierbei, das Vorgehen bei Patienten mit vorbestehender Indikation zur therapeutischen Antikoagulation sowie bei vorbekannter Heparin-induzierter Thrombopenie Typ II (HIT-II) (akut-symptomatisch vs. Jahre zurückliegend) zu eruieren.

**Ergebnisse:**

Die Umfrageergebnisse zeigen, dass auf den meisten der teilnehmenden Intensivstationen eine vorbestehende Antikoagulation größtenteils mit niedermolekularen Heparinpräparaten oder unfraktioniertem Heparin fortgeführt wird. Bei bekannter HIT-II wird Argatroban bevorzugt, unabhängig davon, ob es sich um eine Jahre zurückliegende oder eine akut-symptomatische HIT-II handelt. Eine hohe Variabilität besteht bei der Festlegung der Zielwerte für die Antikoagulation, wobei diese größtenteils deutlich über dem Bereich einer reinen venösen Thromboembolie(VTE)-Prophylaxe zu liegen kommen.

**Schlussfolgerung:**

Die Datenlage zur Fortführung einer über die reine VTE-Prophylaxe hinausgehenden Antikoagulation mit konsekutiv erhöhtem Blutungsrisiko bei Patienten mit Sepsis und septischem Schock ist eingeschränkt, und Therapieentscheidungen unterliegen in vielen Fällen einer individuellen Abwägung des Behandlungsteams. Die Ergebnisse unserer Umfrage implizieren die Notwendigkeit einer systematischen Aufarbeitung dieses Themenfeldes, um die auf vielen Intensivstationen gelebte Praxis mit der gebotenen Evidenz zu unterlegen.

**Zusatzmaterial online:**

Die Online-Version dieses Beitrags (10.1007/s00101-021-01011-9) enthält weitere Tabellen, Abbildungen und Auswertungen der Fragebögen.

Beitrag und Zusatzmaterial stehen Ihnen auf www.springermedizin.de zur Verfügung. Bitte geben Sie dort den Beitragstitel in die Suche ein oder nutzen Sie den QR-Code auf der ersten Seite des Artikels. Das Zusatzmaterial finden Sie beim Beitrag unter „Ergänzende Inhalte“.

## Abstract (en)

**Background:**

A pre-existing anticoagulation treatment and predisposing diseases for thromboembolic events represent common problems in patients with sepsis or septic shock; however, these conditions are not addressed in current national guidelines for sepsis and septic shock. One of the aims of this nationwide survey in Germany was therefore to determine how intensive care physicians deal with these problems.

**Methods:**

From October 2019 to May 2020, we conducted a nationwide survey among German medical directors of intensive care units (ICU) addressing anticoagulation and drug-based prophylaxis of venous thromboembolism (VTE) in patients with sepsis and sepsis-induced coagulopathy. One focus was the procedure for patients with a pre-existing anticoagulation treatment or a previously known heparin-induced thrombocytopenia (HIT) type 2 (acute symptomatic vs. dating back years).

**Results:**

In most of the participating ICUs pre-existing anticoagulation is largely continued with low molecular weight heparin preparations or unfractionated heparin. In patients with pre-existing HIT type 2 both acute symptomatic and dating back years, argatroban represents the drug of choice. There is a high degree of variability in the definition of the target values, usually being well above the range for pure VTE prophylaxis.

**Conclusion:**

Data on the continuation of anticoagulation beyond VTE prophylaxis with a subsequently increased risk of bleeding in patients with sepsis and septic shock is limited and treatment decisions are in many cases subject to individual consideration by the practitioner. The results of our survey imply the need for a systematic work-up of this topic in order to support daily practice in many ICUs with the required evidence.

Obwohl eine vorbestehende Antikoagulation wie auch für thromboembolische Ereignisse prädisponierende Vorerkrankungen häufige Probleme bei der Behandlung von Patienten mit Sepsis darstellen, werden die Herausforderungen im Umgang mit dieser Konstellation von den aktuellen nationalen Leitlinien für Sepsis und septischen Schock ausgeklammert [2, 7]. Sowohl die Surviving Sepsis Campaign (SSC) als auch die Deutsche Sepsis Gesellschaft (DSG) empfehlen in ihren Behandlungsleitlinien lediglich allgemein eine medikamentöse Prophylaxe einer venösen Thromboembolie (VTE) [2, 7]. Die DSG empfiehlt hierfür entweder unfraktioniertes Heparin (UFH) oder niedermolekulare Heparine (NMH). Unklar ist dabei allerdings, wie mit Patienten umzugehen ist, die vorbestehend eine Indikation für eine therapeutische Antikoagulation haben und z. B. auf Vitamin-K-Antagonisten oder sog. direkte orale Antikoagulanzien (DOAK) eingestellt sind. Ein weiteres Problem stellen Patienten mit der Vordiagnose einer Heparin-induzierten Thrombopenie Typ II (HIT-II) dar. Ein Ziel der hier vorgestellten Umfrage war es daher, eine Übersicht über die gelebte Praxis im Umgang mit diesen Patienten auf Intensivstationen in Deutschland zu erhalten.

## Material und Methoden

Bei der hier vorliegenden Arbeit handelt es sich um die Auswertung einer deutschlandweiten Umfrage zum Thema „Antikoagulation und medikamentöse Thromboseprophylaxe“ bei Sepsis und Sepsis-induzierter Koagulopathie (SIC) sowie COVID-19-induzierter Koagulopathie (CAC), die zwischen Oktober 2019 und April 2020 durchgeführt wurde [8]. Im Fokus der hier vorliegenden Analyse stehen die Themenkomplexe „Vorgehen bei Patienten mit vorbestehender Indikation für eine Antikoagulation“ sowie „Vorgehen bei bekannter Diagnose einer HIT-II“ (akut-symptomatisch vs. Jahre zurückliegend). Eine detaillierte Beschreibung der Methodik einschließlich der biometrischen Auswertstrategie kann der Primärpublikation der Umfrage [8] entnommen werden.

Kurz zusammengefasst wurden in der Umfrage die ärztlichen Leiter*Innen einer Intensivstation (ITS) oder eines Intensivbereiches adressiert mit dem Ziel, einen ausgefüllten Fragebogen pro Intensivbereich zu beantworten. Die Teilnahme erfolgte anonym, eine Mehrfachteilnahme war technisch nicht blockiert. Der Web-Link zu dem Fragebogen wurde Oktober 2019 und April 2020 über die Kliniken der SepNet-Studiengruppe an weitere Krankenhäuser unterschiedlicher Versorgungsstufen weitergeleitet. Im April 2020 wurden zusätzlich alle in der Deutschen Interdisziplinären Vereinigung für Intensiv- und Notfallmedizin (DIVI) organisierten intensivmedizinischen Abteilungen über den DIVI-E-Mail-Verteiler zur Teilnahme an der Umfrage eingeladen. Darüber hinaus wurde die Einladung zur Teilnahme im Mai 2020 über den E‑Mail-Verteiler der Interdisziplinären Arbeitsgemeinschaft für Klinische Hämotherapie (IAKH) verschickt. Am 31.05.2020 wurde der Link offline genommen und die Umfrage damit geschlossen.

Kernelement des Fragebogens, der in Zusammenarbeit mit der SepNet Study Group entworfen wurde, waren 2 Fallvignetten: Die erste schilderte einen Fall einer pneumogenen Sepsis, die zweite den einer abdominellen Sepsis bei sekundärer Peritonitis 2 Tage nach erfolgter chirurgischer Fokussanierung. Einfach- und Mehrfachauswahlfragen sowie Freitextfelder wurden zur Erhebung genutzt, und es wurden unter anderem die Fragenkomplexe 1. „*Infrastruktur*“, 2. „*Status quo Antikoagulation*“ und 3. „*Status quo Sepsis*“ erfasst [8]. Neben auswählbaren Antwortmöglichkeiten standen Freitextfelder zur Verfügung.

### Statistik

Die statistische Auswertung erfolgte Software-gestützt mittels deskriptiver Methoden [8]. Verwendet wurde Microsoft® Office Excel (Excel für Mac Version 16.3, Microsoft Corporation, Redmond, WA, USA) sowie Prism® 8 for MacOS, (GraphPad Prism® für Mac Version 8.3.0; GraphPad Software LLC, San Diego, CA, USA).

## Ergebnisse

Insgesamt beantworteten 67 Leiter*Innen von Intensivbereichen unseren Online-Fragebogen. Die Details zur Struktur der teilnehmenden Intensivstationen sind bereits anderweitig veröffentlicht [8]. In Summe gaben die Teilnehmer an, für 1485 Intensivbetten verantwortlich zu sein, an denen eine invasive Beatmung durchgeführt werden kann. Knapp 50 % der Teilnehmer (*n* = 32), waren Leiter von Intensivbereichen in Universitätskliniken, davon 22 Anästhesisten, 6 Internisten, 3 Allgemein‑/Viszeralchirurgen und eine Pädiater*In. Der zweitgrößte Anteil der Teilnehmer waren Leiter von Intensivstationen an akademischen Lehrkrankenhäusern (*n* = 27, davon *n* = 25 Anästhesiologie, *n* = 2 Innere Medizin). Zudem haben *n* = 7 Leiter von Intensivstationen (davon *n* = 6 Anästhesiologie, *n* = 1 Innere Medizin) an Krankenhäusern geantwortet, die kein Lehrkrankenhaus sind [8]. Die meisten der teilnehmenden Intensivstationen gehören zu großen Krankenhäusern mit ≥ 500 Betten (44/67; 65,6 %). Insgesamt 20 Teilnehmer (29,8 %) gaben an, ≥ 250 Fälle von Sepsis und septischem Schock pro Jahr auf ihren Intensivstationen zu behandeln.

### Antikoagulation bei Sepsis mit bereits vorbestehender Indikation zur Vollantikoagulation.

In einer Variation der Fallbeispiele von pneumogener und abdomineller Sepsis wurde nach dem Vorgehen bei Patienten gefragt, bei denen eine Antikoagulation mit direkten oralen Antikoagulanzien (DOAK; wie z. B. Faktor-Xa-Inhibitoren Apixaban [Eliquis®, Bristol-Myers Squibb, New York, USA, und Pfizer Pharma, New York, USA], Edoxaban [Lixiana®, Daiichi Sankyo Europe, Tokyo, Japan], Rivaroxaban [Xarelto®, Bayer AG, Leverkusen, Deutschland] oder der Thrombininhibitor Dabigatran[etexilat] [Pradaxa®, Boehringer Ingelheim, Ingelheim am Rhein, Deutschland]) oder Vitamin-K-Antagonisten (z. B. Marcumar®, MEDA Pharma, Bad Homburg, Deutschland) wegen Vorhofflimmerns oder als Sekundärprophylaxe bei einer zurückliegenden tiefen Beinvenenthrombose (TVT) oder Lungenarterienembolie (LAE) bestand.

**Figure 1:**
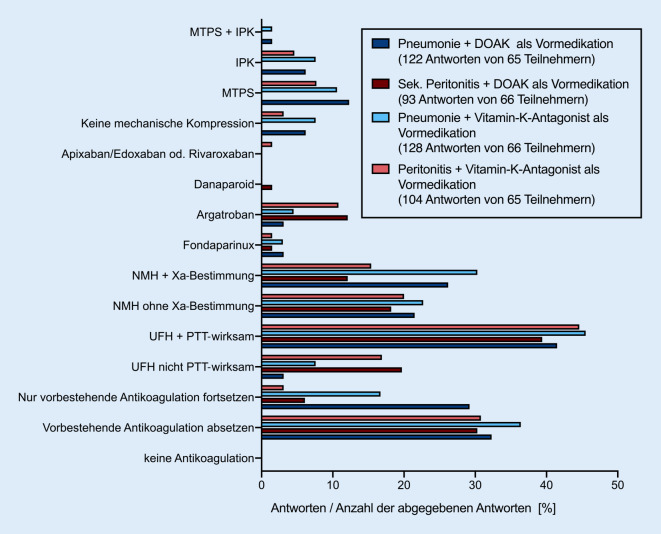

Insgesamt 22 (33,8 %) der 65 Teilnehmenden, die eine Antwort auf diese Frage abgaben, würden im Falle einer pneumogenen Sepsis eine vorbestehende Antikoagulation mit DOAKs bei vorbestehendem VHF absetzen. Im Falle einer abdominellen Sepsis würden dies 20 von 66 (30,3 %) tun (Abb. 1; Online-Zusatzmaterial (ESM) 1 – Tabellarische Übersicht der bei Sepsis verwendeten Antikoagulation bei vorbestehender Antikoagulation).

**Figure 2:**
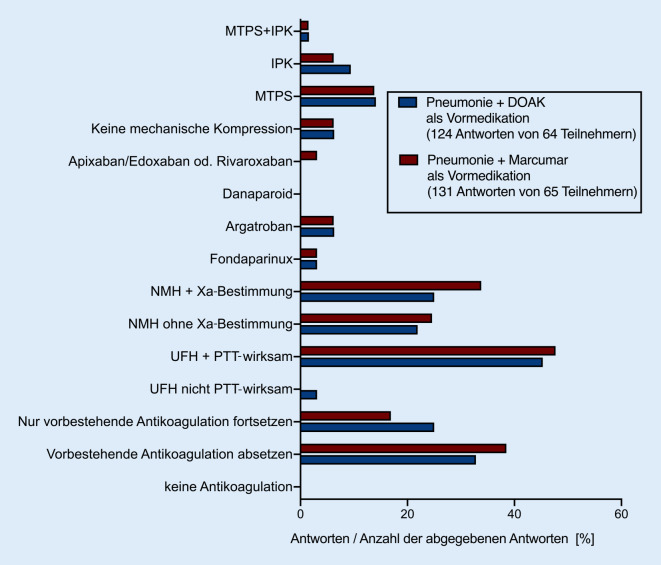

**Figure 3:**
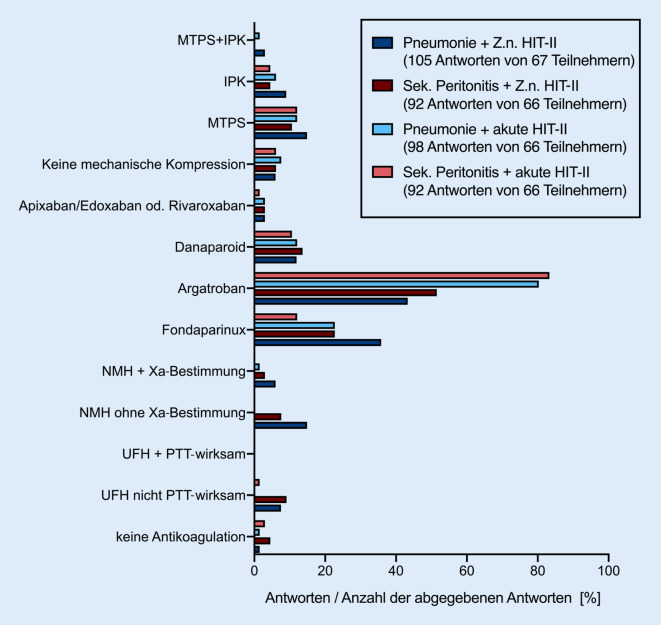

Ähnlich verhält es sich, wenn die Indikation zur Antikoagulation eine länger zurückliegende tiefe Beinvenenthrombose (TVT) oder eine Lungenarterienembolie (LAE) ist (21/64, 32,8 %; Abb. 2, ESM 1 – Tabellarische Übersicht der bei Sepsis verwendeten Antikoagulation bei vorbestehender Antikoagulation). Im Falle von Vitamin-K-Inhibitoren (Marcumar®) als Vormedikation würde diese bei pneumogener Sepsis auf 24 von 66 (36,4 %) und bei abdomineller Sepsis auf 20 von 65 (30,8 %) der ITS abgesetzt werden. Bei lange zurückliegender TVT und LAE würden Vitamin-K-Inhibitoren sogar noch etwas häufiger, auf 38,5 % der ITS, abgesetzt werden (25/65, 38,5 %; Abb. 3).

Stattdessen gaben unabhängig vom Fokus der Sepsis (pneumogene Sepsis oder abdominelle Sepsis) und der Vormedikation etwa 40–47 % der Teilnehmenden an, die Vollantikoagulation während des Aufenthaltes auf der ITS mittels PTT-gesteuerter Gabe von UFH aufrechtzuerhalten (Abb. 3). Interessanterweise geben jedoch auch 20 % der Antwortenden unabhängig von dem Sepsisfokus und der Vormedikation an, eine nicht FXa-kontrollierte Vollantikoagulation mit NMH durchzuführen. Auf etwa 25 % der ITS wird NMH bei pneumogener Sepsis FXa-kontrolliert eingesetzt, während dies in 12–15 % der Fälle bei abdomineller Sepsis erfolgt. Bei pneumogener Sepsis würden 29,3 % der Befragten die Vormedikation zur Antikoagulation fortführen, wenn es sich um ein DOAK handelt. Im Falle von Vitamin-K-Inhibitoren und pneumogener Sepsis würden dies immerhin noch 16,7 % tun.

Anders sieht es bei der abdominellen Sepsis aus; hier würden nur 6,1 % eine Vormedikation mit einem DOAK und 3,1 % eine Vormedikation mit Vitamin-K-Inhibitoren fortführen. Eine mechanische VTE-Prophylaxe wird bei bestehender Vormedikation in weniger als 20 % der Antworten als Option genannt. Auch in den Freitextantworten zum Umgang mit vorbestehender Antikoagulation fand sich eine hohe Variabilität bezüglich der eingesetzten Dosierungen und Zielwerte. Die ausführlichen Freitextantworten sind in den Internet-Supplements (ESM 2 – Freitextantworten zur Fortführung der Antikoagulation bei vorbestehender Behandlung mit DOAKs; ESM 3 – Freitextantworten zur Fortführung der Antikoagulation bei *vorbestehender Behandlung* mit Marcumar bei VHF; ESM 4 – Vorbestehende Antikoagulation bei TVT) beinhaltet.

### VTE-Prophylaxe und Antikoagulation akuter HIT-II versus Jahre zurückliegender HIT-II.

In weiteren Variationen der oben genannten Fallbeispiele von pneumogener und abdomineller Sepsis wurde nach dem Vorgehen zur VTE-Prophylaxe bei einer in der Krankengeschichte (Zustand nach [Z. n.] vor Jahren) beschriebenen HIT-II sowie nach der Antikoagulation bei einer akut-symptomatischen HIT-II gefragt. Unabhängig davon, ob eine klinisch apparente HIT-II Jahre zurückliegt und damit gemäß Leitlinie eine reine VTE-Prophylaxe indiziert ist oder eine HIT-II akut aufgetreten und symptomatisch ist, sodass eine Antikoagulation indiziert ist, gab die Mehrzahl der Teilnehmenden an, Argatroban (Argatra®, Mitsubishi Tanabe Pharma Europe Ltd., London, Vereinigtes Königreich) bei derartigen Patienten einzusetzen (Abb. 3, ESM 5 – Tabelle: Antikoagulation bei Sepsis und HIT-II [akut-symptomatisch vs. lange zurückliegend]). Bei einer akuten HIT-II gaben 4 von 5 der Befragten an, Argatroban zu nutzen (pneumogene Sepsis + HIT-II *n* = 53, 80,3 %; AS + HIT-II *n* = 55, 83,3 %), bei einer Jahre zurückliegenden HIT-II war es immerhin noch jeder Dritte (pneumogene Sepsis + Z. n. HIT-II *n* = 29, 43,3 %; abdominelle Sepsis + Z. n. HIT-II *n* = 34, 51,5 %). Als Alternative wurde insbesondere Fondaparinux (Arixtra®, Aspen Pharma Trading Limited, Dublin, Irland; pneumogene Sepsis + HIT-II *n* = 15, 22,7 %; abdominelle Sepsis + HIT-II *n* = 8, 12,1 %; pneumogene Sepsis + Z. n. HIT-II *n* = 24, 35,8 %; AS + Z. n. HIT-II *n* = 15, 22,7 %) genannt. Jeweils knapp 10–15 % der Teilnehmer gaben an, bei jeder der genannten Konstellationen (abdominelle Sepsis oder pneumogene Sepsis + akute oder zurückliegende HIT-II) von Sepsis und HIT-II auch Danaparoid (Orgaran®, Aspen Pharma Trading Limited, Dublin, Irland) zu nutzen (Abb. 3, ESM 6 – Freitextantworten zur Antikoagulation bei vorbestehender HIT-II). Apixaban, Edoxaban und Rivaroxaban kommen in den genannten Situationen auf etwa 3 % der ITS zum Einsatz. Eine mechanische VTE-Prophylaxe wird auf 10–15 % der Intensivstationen in Form von MTPS und in 4,5–9 % in Form von IPK durchgeführt (Abb. 3, ESM 5 – Tabelle: Antikoagulation bei Sepsis und HIT-II [akut-symptomatisch vs. lange zurückliegend]).

### Freie Kommentare zur VTE-Prophylaxe bei zurückliegender HIT-II.

Sowohl bei pneumogener als auch bei abdomineller Sepsis gaben die meisten Teilnehmer im Rahmen von Freitextkommentaren an, im Falle einer Jahre zurückliegenden HIT-II Argatroban mit PTT-Zielwerten von < 40 s oder zwischen 40 und 60 s zu nutzen. In Intensivbereichen, in denen Fondaparinux genutzt wird, geschieht dies mit der empfohlenen prophylaktischen Dosierung von 2,5 mg/Tag. In Einzelfällen wurden auch andere Medikamente und Dosierungen genannt (vgl. ESM 6 – Freitextantworten zur Antikoagulation bei vorbestehender HIT-II).

### Freie Kommentare zur Antikoagulation bei akuter HIT-II.

Im Falle einer akuten HIT-II bei pneumogener Sepsis unterschieden sich die Freitextantworten deutlich stärker: In 16 von 21 Freitextantworten wurde Argatroban genannt. Dabei gab ein Teilnehmer an, Argatroban „nicht PTT-wirksam“ zu verwenden, und 4 antworteten „nach PTT“, ohne einen konkreten Zielbereich zu benennen. Einmal wurde ein PTT-Zielwert von 25–40 s angegeben, 1‑mal 45 s, 1‑mal 50 s, 3‑mal 60 s, 1‑mal 50–60 s, 1‑mal 50–70 s und 3‑mal 60–80 s. Fondaparinux wurde in diesem Fall 2‑mal in der Dosierung von 7,5 mg/Tag und 3‑mal in der Dosierung 2,5 mg/Tag genannt. Auch in den Freitextantworten für den Fall einer akuten HIT-II bei abdomineller Sepsis streuten die Zielwerte für die PTT vergleichbar stark (zwischen „nicht PTT-wirksam“ und „60–70 s“). Auch in diesem Fall wird Fondaparinux sowohl in prophylaktischer als auch in therapeutischer Dosierung verwendet. Interessanterweise wurde für diesen Fall in einem Freitextkommentar UFH (500–800 IE/h) mit einer Ziel-PTT von 35 s genannt. Die ausführlichen Freitextkommentare zu den 4 Fallvignetten mit HIT-II können im ESM 6 – Freitextantworten zur Antikoagulation bei vorbestehender HIT-II nachgelesen werden.

## Diskussion

Ziel der hier vorliegenden Auswertung der deutschlandweiten Umfrage war es zu eruieren, wie Intensivmediziner mit den Problemen einer vorbestehenden Antikoagulation sowie der Thematik einer akut-symptomatischen vs. lange zurückliegenden HIT-II bei der Behandlung von Patienten mit Sepsis und septischem Schock umgehen. Da hierbei nicht die gesamte Heterogenität der Indikationen für eine Antikoagulation bzw. nicht differenziert für unterschiedliche thrombophile Diathesen abgebildet werden konnte, lag der Fokus exemplarisch 1. auf dem Umgang mit vorbestehender Antikoagulation bei Vorhofflimmern sowie 2. bei Sekundärprophylaxe nach einer zurückliegenden TVT mit/ohne LAE. Der dritte Fokus lag auf dem Procedere bei septischen Patienten mit akut-symptomatischer vs. lange zurückliegender HIT-II: In dieser Patientengruppe ist eine Kontraindikation gegen die in den Sepsis-Leitlinien empfohlenen Medikamente zur VTE-Prophylaxe anzunehmen.

Die in der Befragung gewählten Krankheitsbilder haben eine relevante klinische Bedeutung, zumal bei knapp 20 % der Patienten mit Sepsis oder septischem Schock ein Vorhofflimmern vorbesteht [11] und sich dieser Anteil in den kommenden Jahren im Zuge der demografischen Entwicklung noch wahrscheinlich erhöhen wird. Die Inzidenz einer HIT-II wird mit 1 pro 5000 hospitalisierten Patienten geschätzt, während dies in einzelnen Kollektiven (wie z. B. kardiochirurgischen Patienten) allerdings auch deutlich höher sein kann. Hinzu kommen Patienten, die im Rahmen zurückliegender Krankenhausaufenthalte eine HIT entwickelt hatten. Die medikamentöse Thromboseprophylaxe und Antikoagulation stellt in dieser Patientengruppe jedoch eine besondere Herausforderung dar, da gerade bei dieser Patientengruppe die Gefahr arterieller Embolien deutlich erhöht ist [4, 5].

Ziel unserer Umfrage war es, ein deutschlandweites Bild des Umgangs mit den vorgenannten Patientenpopulationen zu erheben. Hinsichtlich der Repräsentativität unserer Umfrage ist von Bedeutung, dass die teilnehmenden 67 Leiter von Intensivbereichen die Verantwortung für 20,5 % der 7230 „High-Care-Betten“ tragen, die aktuell von der DIVI in Deutschland registriert sind [6]. Bei insgesamt 35 Universitätsklinika in Deutschland [9] sowie basierend auf der Annahme, dass es einen anästhesiologischen Intensivbereich pro Universitätsklinikum gibt oder nur einer pro Universitätsklinikum geantwortet hat, kann davon ausgegangen werden, dass mindestens zwei Drittel (22/35 = 62,8 %) der Universitätsklinika in unserem Kollektiv erfasst sind.

Unsere Ergebnisse bezüglich des Umgangs mit vorbestehender Antikoagulation bei Vorhofflimmern unterscheiden sich von Daten, die aus den USA vorliegen. Aj et al. beschreiben dabei nach einer Auswertung von etwa 38.600 Patienten mit Sepsis und septischem Schock mit der Begleitdiagnose VHF (aber ohne anderen Grund für eine Antikoagulation), dass nur etwa 35,3 % der Patienten eine intravenöse Antikoagulation erhielten, die über eine reine VTE-Prophylaxe hinausging [1]. Unsere Daten sprechen dafür, dass in Deutschland vergleichsweise häufiger eine therapeutische Antikoagulation während des Intensivaufenthaltes angestrebt wird. Unsere Daten lassen zudem annehmen, dass dabei insbesondere im Rahmen eines pneumogenen Fokus die Dauermedikation weitergeführt oder diese durch den PTT-wirksamen Einsatz von UFH bzw. den Anti-Xa-wirksamen Einsatz von NMH ersetzt wird. Ob sich ein solches Vorgehen tatsächlich vorteilhaft für die Patienten auswirkt, ist bislang unklar. So deuten die Daten von Aj et al. darauf hin, dass eine über die VTE-Prophylaxe hinausgehende Antikoagulation bei Patienten mit Sepsis und Vorhofflimmern das Risiko eines ischämischen Schlaganfalls zumindest kurzfristig (während der untersuchten Krankenhausaufenthalte) nicht zu senken vermag (1,3 % vs. 1,4 % im Vergleich zu einer mittels Propensity-Matching erstellten Vergleichskohorte), gleichzeitig aber das Risiko relevanter Blutungen erhöht (8,6 % vs. 7,2 % RR, 1,21; 95 % CI, 1,10–1,32) [1]. Dies ist insofern interessant, als dass unseren Daten zufolge in Deutschland auch bei der relativen Indikation zu einer Antikoagulation aufgrund einer Jahre zurückliegenden LAE noch eine starke Tendenz zu einer Fortführung der Antikoagulation während der intensivmedizinischen Behandlung im Rahmen einer Sepsis besteht. Entgegen den aktuellen Leitlinien besteht also in Deutschland die Tendenz, eine vorbestehende Antikoagulation auch während der intensivmedizinischen Behandlung einer Sepsis fortzusetzen. Angesichts der verfügbaren Evidenz [1] sollte dieses Vorgehen allerdings kritisch diskutiert und ggf. modifiziert werden.

Der zweite Fokus unserer Untersuchung lag auf dem Handling von Patienten mit akut-symptomatischer vs. lange zurückliegender HIT-II. Entsprechend aktueller Leitlinien, gaben die meisten Antwortenden an, Argatroban im Falle einer akut-symptomatischen HIT-II zu verwenden. Interessant ist, dass auch hierbei das Therapieziel nicht einheitlich ist. Dabei führen einige Intensivbereiche bei diesen Patienten eher eine medikamentöse VTE-Prophylaxe mit PTT-Zielwerten < 40 s durch, während andere deutlich darüber liegen. Obwohl die bei schweren Formen der HIT-II auftretende disseminierte Koagulopathie es aus pharmakologischen Überlegungen sinnvoll erscheinen lässt, bei HIT-II eine über die reine VTE-Prophylaxe hinausgehende Antikoagulation durchzuführen, ist der therapeutische Nutzen eines solchen Vorgehens bis heute nicht durch Studien belegt. Mit Argatroban nutzen die meisten Intensivbereiche das Medikament, welches von der European Society of Anesthesia and Intensive Care (ESAIC) bei Patienten mit (bei kritisch kranken Patienten häufiger) Niereninsuffizienz derzeit als Mittel der ersten Wahl bei akuter HIT-II eingestuft wird [3]. Bei Leberinsuffizienz muss allerdings eine Dosisanpassung und bis zur Erreichung eines „Steady-States“ ein engmaschiges Monitoring der PTT erfolgen! Darüber hinaus ist Argatroban formal nur für eine therapeutische Antikoagulation bei nachgewiesener HIT-II zugelassen. Der Einsatz von Argatroban zur reinen VTE-Prophylaxe bei Z. n. lange zurückliegender HIT-II ohne aktuell bestehende Thrombose- oder Emboliezeichen erfolgt als Off-label-Use.

Auch der Einsatz von Fondaparinux und Danaparoid, den einige der Antwortenden berichten, steht bei akut-symptomatischer HIT-II in Einklang mit der ESAIC-Leitlinie [3]; allerdings unter der Einschränkung, dass in 5–10 % eine Kreuzsensibilisierung vorliegen kann [10].

Im Vergleich der Fachdisziplinen Anästhesiologie und Innere Medizin (Vergleiche zu und zwischen anderen Fachdisziplinen sind aufgrund der geringen Zahl an Antworten nicht aussagekräftig) zeigten sich u. a. orientierend für die Innere Medizin ein höherer Anteil der Behandlung mit NMH mit Anti-Xa-Bestimmung bei Pneumonie und DOAK als Vormedikation, ein höherer Anteil der Behandlung mit UFH (PTT-wirksam) bei abdomineller Sepsis und DOAK in Vormedikation, ein höherer Anteil der Behandlung mit UFH (PTT-wirksam) bei abdomineller Sepsis und Marcumar als Vormedikation sowie ein höherer Anteil der Anwendung von Danaparoid bei HIT-II (s. ESM 7 – Antikoagulation auf internistisch vs. anästhesiologisch geführten ITS bei vorbestehender Antikoagulation mit DOAK bei Vorhofflimmern; ESM 8 – Antikoagulation auf internistisch vs. anästhesiologisch geführten ITS bei vorbestehender Antikoagulation mit Marcumar bei VHF; ESM°9 – Antikoagulation auf internistisch vs. anästhesiologisch geführten ITS bei akuter Heparin-induzierter Thrombozytopenie Typ 2).

Eine Limitation unserer Datenerhebung stellt das Fehlen von Detailinformation hinsichtlich des spezifischen Umgangs mit Zielwerten wie Anti-Xa sowie PTT dar. Es ist jedoch anzunehmen, dass Zielbereiche im Anti-Xa-Monitoring wie auch Abnahmezeitpunkte (Tal- oder Spitzenspiegel) sowie das Vorgehen beim PTT-Monitoring in verschiedenen Zentren und Kliniken durchaus unterschiedlich definiert sind bzw. gehandhabt werden, was die in unserer Studie beobachtete Variabilität in der klinischen Praxis noch unterstreicht. Wie bereits ausgeführt [8], sind bei der Therapiesteuerung durch die PTT zudem als weiter komplizierende Faktoren unterschiedliche laborchemische Verfahren wie auch bekannte potenzielle Störfaktoren der Messung beim Patienten mit Sepsis [12–14] zu diskutieren, welche aus Sicht der Autoren noch zu einer höheren Divergenz zwischen einzelnen Kliniken und Zentren beitragen können.

In der Zusammenschau zeigen unsere Ergebnisse, dass es in Deutschland einen klaren Trend gibt, eine vorstehende Antikoagulation bei Patienten mit Sepsis und septischem Schock fortzuführen und nicht auf eine reine VTE-Prophylaxe zu reduzieren. Die vom Patienten als Hausmedikation verwendeten Substanzen werden dabei – im Rahmen der intensivmedizinischen Behandlung einer Sepsis – meist durch Heparine (UFH und NMH) ersetzt werden. Bei der Entscheidung, ob UFH oder NMH zum Einsatz kommen, scheinen dabei weniger die in der Hausmedikation verwendete Substanzklasse oder die primärere Indikation der vorbestehenden Antikoagulation eine Rolle zu spielen, sondern vielmehr das geschätzte Blutungsrisiko und die Gewohnheiten des behandelnden Hauses. Relevante Unterschiede zwischen einzelnen Häusern zeigen sich dabei sowohl bei der Auswahl des Heparin(oids) als auch bei der Festlegung der laborchemischen Zielwerte für die Antikoagulation.

Unsere Ergebnisse zeigen, dass die Frage nach den Risiken und dem Nutzen einer Fortsetzung einer therapeutischen Antikoagulation unter Intensivtherapie einer systematischen Aufarbeitung bedarf und dabei v. a. geeignete Zielwerte einer Therapie adressiert werden müssen. Hierbei muss es insbesondere darum gehen, das Risiko von Blutungskomplikationen präziser und, wenn möglich, Patienten-individuell abzuschätzen und gegen das Risiko von Thromboembolien insbesondere im Zusammenhang mit einer Sepsis-induzierten Koagulopathie abzuwägen.

## Supplementary Information




