# Supplementary material for: Practice of pharmaceutical thrombosis prophylaxis and anticoagulation in patients with sepsis and pre-existing anticoagulation or heparin-induced type II thrombocytopenia—Results of a nationwide survey in German intensive care units
Source: Anaesthesist. 2021 Aug 5;71(3):193–200. [Article in German] doi: 10.1007/s00101-021-01011-9 (PMC8340811; doi:10.1007/s00101-021-01011-9)

Zusatzmaterial zum Beitrag „Praxis der medikamentösen Thromboseprophylaxe und Antikoagulation bei Patienten mit Sepsis und vorbestehender Antikoagulation oder Heparin-induzierter Thrombozytopenie Typ II“ von Schmoch, T, Brenner, T, Becker-Pennrich A et al. (2021) in *Der Anaesthesist.*

Beitrag und Zusatzmaterial stehen Ihnen auf www.springermedizin.de zur Verfügung. Bitte geben Sie dort den Beitragstitel in die Suche ein.

ESM 1. Tabellarische Übersicht der bei Sepsis verwendeten Antikoagulation bei vorbestehender Antikoagulation.

	Pneumonie		Sekundäre Peritonitis		Pneumonie + DOAK bei VHF		Peritonitis + DOAK		Pneumonie + DOAK bei (LAE vor Jahren)		Pneumonie + Marcumar bei VHF		Peritonitis + Marcumar		Pneumonie + Marcumar bei LAE vor Jahren	
	n	%	n	%	n	%	n	%	n	%	n	%	n	%	n	%
Anzahl der abgegebenen Antworten	67		66		65		66		64		66		65		65	
Anzahl der Antworten incl. Mehrfachnennungen	102		91		122		93		124		128		104		131	
keine Antikoagulation	1	1,5	1	1,5	0	0,0	0	0,0	0	0,0	0	0,0	0	0,0	0	0,0
Vorbestehende Antikoagulation absetzen	n.a.	n.a.	n.a.	n.a.	21	32,3	20	30,3	21	32,8	24	36,4	20	30,8	25	38,5
nur vorbestehende Antikoagulation fortsetzen	n.a.	n.a.	n.a.	n.a.	19	29,2	4	6,1	16	25,0	11	16,7	2	3,1	11	16,9
UFH nicht PPT-wirksam	23	34,3	34	51,5	2	3,1	13	19,7	2	3,1	5	7,6	11	16,9	0	0,0
UFH + PPT-wirksam	2	3,0	2	3,0	27	41,5	26	39,4	29	45,3	30	45,5	29	44,6	31	47,7
NMH ohne Anti-FXa-Bestimmung	43	64,2	29	43,9	14	21,5	12	18,2	14	21,9	15	22,7	13	20,0	16	24,6
NMH + Anti-FXa-Bestimmung	8	11,9	7	10,6	17	26,2	8	12,1	16	25,0	20	30,3	10	15,4	22	33,8
Fondaparinux	1	1,5	0	0,0	2	3,1	1	1,5	2	3,1	2	3,0	1	1,5	2	3,1
Argatroban	1	1,5	1	1,5	2	3,1	8	12,1	4	6,3	3	4,5	7	10,8	4	6,2
Danaparoid	0	0,0	0	0,0	0	0,0	1	1,5	0	0,0	0	0,0	0	0,0	0	0,0
Apixaban/Edoxaban od. Rivaroxaban	0	0,0	0	0,0	0	0,0	0	0,0	0	0,0	0	0,0	1	1,5	2	3,1
Keine mechanische Kompression	6	9,0	4	6,1	4	6,2	0	0,0	4	6,3	5	7,6	2	3,1	4	6,2
MTPS	10	14,9	9	13,6	8	12,3	0	0,0	9	14,1	7	10,6	5	7,7	9	13,8
IPK	6	9,0	4	6,1	4	6,2	0	0,0	6	9,4	5	7,6	3	4,6	4	6,2
MTPS+IPK	1	1,5	0	0,0	1	1,5	0	0,0	1	1,6	1	1,5	0	0,0	1	1,5

DOAK = direkte orale Antikoagulanzien, IPK = intermittierende pneumatische Kompression, NMH = niedermolekulares Heparin, MTPS = medizinische Thrombose-Prophylaxestrümpfe, PTT= partielle Thromboplastinzeit, UFH = unfraktioniertes Heparin; VHF = Vorhofflimmern.

ESM 2. Freitextantworten zur Fortführung der Antikoagulation bei vorbestehender Behandlung mit DOAKs.

Fallvignette 1: *Nicht operativer Patient, 68 Jahre, Pneumonie, intubiert, beatmet mit FiO2 0,60, kein bis niedriger Katecholaminbedarf (kein weiteres Organversagen, kein Schock) und zusätzlich: orale Antikoagulation mit DOAK bei vorbestehendem chronischem Vorhofflimmern.*

Insgesamt 26 der 65 Teilnehmer, die auf diesen Fall geantwortet haben, gaben zusätzlich Freitextkommentare ab. In fünf Freitextantworten wurde beschrieben, dass DOAKs weiter gegeben werden, solange kein weiteres Organversagen hinzu kommt. In 10 Antworten wurde Enoxaparin „gewichtsadaptiert" mit 1mg/kg Körpergewicht oder in Dosierungen von 60-80mg bis hin zu 2x100mg (ohne Dosisanpassung nach Gewicht) genannt. In 8 Antworten wurde UFH genannt, dabei wurde einmal ein PTT-Zielwert von 40-50s, einmal 50-60s, einmal 60s und viermal 60-80s genannt. Ein Teilnehmer gab nur an, dass eine PTT-Kontrolle durchgeführt wird, jedoch ohne PTT-Zielbereich. Auf einer der teilnehmenden ITS wird in einem solchen Fall Argatroban mit einer Ziel-PTT von 40s eingesetzt. Drei ITS setzen Certoparin ein, zwei dabei in einer Dosierung von 1 x 8.000 I.E. und eine in einer Dosierung von 2 x 8000 IE. In einer Antwort wurde Tinzaparin in einer Dosierung von 175IE/kg KG genannt.

Fallvignette 2: *Abdominelle Sepsis bei sekundärer Peritonitis, am 2. postoperativen Tag bei DOAK als Vormedikation.*

Es wurden 24 Freitextantworten abgegeben. Am häufigsten (13-mal) wurde UFH genannt. Dabei variierten die Antworten von Angaben, die für eine klassische VTE-Prophylaxe sprechen, wie „in prophylaktischer Dosierung", „Heparin 300IE/h", „Heparin 400IE/h" oder „10 mg/24 h" über PTT-Zielwertangaben von 40-50s, bis hin zu Zielwerten einer therapeutischen Antikoagulation (PTT 60-80s). Auch Enoxaparin wurde viermal genannt. Hierbei wurden Dosierungen von 2 x 40mg/d, 2 x 1mg/kg KG und 2 x 70-80mg/d angegeben. Auch Argatroban kommt mit PTT-Zielwerten von 40-60s (eine Nennung) oder 50-60s (eine Nennung) zum Einsatz. Andere Teilnehmer nannten Certoparin in Dosierungen von 1 x 3.000 I.E.; 2 x 3.000 I.E. oder 1 x 8.000 I.E.. In einer Freitextantwort wurde angegeben, dass in einem solchen Fall die Vormedikation mit DOAKs fortgeführt wird.

ESM 3. Freitextantworten zur Fortführung der Antikoagulation bei vorbestehender Behandlung mit Marcumar bei VHF.

Fallvignette 3: *Pneumogene Sepsis, orale Antikoagulation mit Marcumar bei vorbestehendem chronischen Vorhofflimmern.*

Zu diesem Fall wurden insgesamt 28 Freitextantworten abgegeben. Einige Teilnehmer machten dabei Angaben zu mehreren Antikoagulationsoptionen. Drei Antwortende gaben an, Vitamin-K-Antagonisten weiter zu führen. Dabei wurden als Ziel International Normalized Ratio (INR)-Werte zwischen 1,8 und 2,2 bzw. >2 genannt. Elf Teilnehmende machten Angaben zu Ihrer Verwendung von Enoxaparin: Die Dosisangaben variierten dabei zwischen „2 x 100 IE/kg“, “2 x tgl. 0,01 ml x kgKG”, “Clexane 2 x 60-80 mg“, “2x0,7 0,8 mg/d s.c.“ und „nach KG“. Als Anti-FXa-Zielwerte wurden dabei Werte von „0,2-0,4 IE/ml“ bis „0,4-1,0 IE/ml“ genannt. In einer der teilnehmenden Kliniken wird in einem solchen Fall Argatroban mit einer Ziel-PTT von 60-80s verwendet. In acht Freitextkommentaren wurde UFH genannt. Dabei wurden Ziel-PTT-Werte von 40s, 40-50s (2x), 60s und 60-80s aufgeführt. Drei der Teilnehmenden nannten Certoparin in Dosierungen von 1x oder 2 x 8.000 I.E./d)

Fallvignette 4: *Abdominelle Sepsis: am 2. postoperativen Tag bei Vitamin-K-Antagonist als Vormedikation bei vorbestehendem chronischen Vorhofflimmern.*

In den 22 abgegebenen Freitextkommentaren wurden 9 Angaben zur Verwendung von UFH gemacht. Dabei variierten die Dosisangaben von 12 I.E./kg/h (Ziel-PTT 60s) über 20 I.E./kg/h oder 1200-1400 I.E./h oder 500-800 I.E./h „unter PTT-Kontrolle“. Die angegebenen PTT-Zielwerte streuten zwischen 40s und 80s. Fünf Antwortende machten Angaben zur Verwendung von Enoxaparin. Dabei variierten die genannten Dosierungen zwischen „40 mg s.c. 1-0-1“, „1mg/kg KG 1-0-1“, „2 x 0,7-0,8 mg/d s.c.“ und „2 x tgl. n. kgKG“. In einer Antwort wurde ein Anti-FXa-Ziel-Talspiegel von 0,2 -0,4 IE/ml angegeben. In zwei Antworten wurde Argatroban mit einer Ziel-PTT von 40-60 s bzw. 50-60s genannt. In drei Antworten wurde Certoparin aufgeführt, mit Dosierungen von „3.000 I.E.“ 1x tgl., „3.000 I.E. 2x tgl.“ und „8.000 I.E. 1x tgl.“.

ESM 4. Vorbestehende Antikoagulation bei TVT.

Fallvignette 5: *Pneumogene Sepsis + vorbestehend orale Antikoagulation mit DOAK bei Z.n. mehrfachen tiefen Beinvenenthrombosen und Lungenarterienembolie vor Jahren.*

In fünf Freitextkommentaren gaben die Teilnehmer an, DOAKs (weiter) zu verwenden. In zwei dieser 5 Antworten wurde spezifiziert, dass Apixaban (5mg 1-0-1) eingesetzt würde. In neun Antworten wurde Enoxaparin genannt. Wie auch in den anderen Fällen variierten die Dosisangaben erheblich. Es wurden Anti-FXa-Zielwerte von 0,1 – 1,0 U/ml genannt. Ähnlich weit war die Streuung bei den Angaben zu UFH (9 Antworten). Auch hier variierten die PTT-Zielwerte zwischen 40s und 80s. Es gab eine Angabe zu Argatroban (Ziel-PTT 60-80s) und 3 Nennungen von Certoparin, einmal in der Dosierung 1x8.000 I.E./d, einmal 1-2x 8.000 I.E./d und einmal 2x8.000 I.E./d.

Fallvignette 6: *Pneumogene Sepsis + vorbestehend orale Antikoagulation mit Vitamin-K-Antagonisten bei Z.n. mehrfachen tiefen Beinvenenthrombosen und Lungenarterienembolie vor Jahren.*

Es wurden 27 Freitextantworten abgegeben. In 3 der teilnehmenden Intensivbereichen wird Vitamin-K-Antagonisten fortgeführt (Ziel INR 1,8-2,0 bzw. >2). Ansonsten entsprachen die genannten Zielwerte für Enoxaparin und UFH weitgehend den Angaben zu Fallvignette 5. Auch die genannten Dosisschemata für Certoparin wiesen die gleiche Streuung wie bei Fallvignette 5 auf.

ESM 5. Tabelle: Antikoagulation bei Sepsis und HIT-II (akut-symptomatisch vs. lange zurückliegend).

	Pneumonie + Z.n. HIT-II		Pneumonie + akute HIT -II		Peritonitis + Z.n. HIT-II		Peritonitis + akute HIT-II	
	n	%	n	%	n	%	n	%
Anzahl der abgegebenen Antworten	67		66		66		66	
Anzahl der Antworten incl. Mehrfachnennungen	105		98		92		92	
keine Antikoagulation	1	1,5	1	1,5	3	4,5	2	3,0
Vorbestehende Antikoagulation absetzen	0	0,0	0	0,0	0	0,0	0	0,0
nur vorbestehende Antikoagulation fortsetzen	0	0,0	0	0,0	2	3,0	3	4,5
UFH nicht PPT-wirksam	5	7,5	0	0,0	6	9,1	1	1,5
UFH + PPT-wirksam	0	0,0	0	0,0	0	0,0	0	0,0
NMH ohne Anti-FXa-Bestimmung	10	14,9	0	0,0	5	7,6	0	0,0
NMH + Anti-FXa-Bestimmung	4	6,0	1	1,5	2	3,0	0	0,0
Fondaparinux	24	35,8	15	22,7	15	22,7	8	12,1
Argatroban	29	43,3	53	80,3	34	51,5	55	83,3
Danaparoid	8	11,9	8	12,1	9	13,6	7	10,6
Apixaban/Edoxaban od. Rivaroxaban	2	3,0	2	3,0	2	3,0	1	1,5
Keine mechanische Kompression	4	6,0	5	7,6	4	6,1	4	6,1
MTPS	10	14,9	8	12,1	7	10,6	8	12,1
IPK	6	9,0	4	6,1	3	4,5	3	4,5
MTPS+IPK	2	3,0	1	1,5	0	0,0	0	0,0

DOAK = direkte orale Antikoagulanzien, HIT-II = Heparin-induzierte Thrombozytopenie Typ II, IPK = intermittierende pneumatische Kompression, NMH = niedermolekulares Heparin, MTPS = medizinische Thrombose-Prophylaxestrümpfe, PTT= partielle Thromboplastinzeit , UFH = unfraktioniertes Heparin; VHF = Vorhofflimmern.

ESM 6 Freitextantworten zur Antikoagulation bei vorbestehender HIT-II

Fallvignette 7: *Pneumogene Sepsis und HIT-II in der Anamnese, Jahre zurückliegend, aktuell kein Hinweis auf thromboembolische Ereignisse oder Thrombozytenabfall.*

In 8 Freitextfeldern wurde Argatroban mit PTT-Zielwerten zwischen 40 und 60s genannt. Zwei Teilnehmer gaben Dosierungen an, die bei 0,25 I.E./kgKG/h und 0,5 µg/kg KG/min lagen. Einmal wurde angegeben, Argatroban „nicht-PTT-wirksam“ zu verwenden. Insgesamt 13 Antwortende gaben Fondaparinux (Arixtra®) in prophylaktischer Dosierung (1x2,5 mg) an. In einer Antwort wurde angegeben, dass neben Argatroban auch Dabigatran und Danaparoid zum Einsatz kommen, „Fondaparinux [jedoch] nur in ausgewählten Indikation[en]“. 2 x Tinzaparin (Innohep®) 4.500 IE 1x/d s.c, 2 x Enoxaparin 40 mg s.c., 2 x Certoparin (Mono-Embolex®) 3.000IE 1x tgl. Jeweils einer der teilnehmenden Leiter einer ITS gab an, Danaparoid 2 x 750IE s.c. oder Apixaban 2,5 mg p.o. 1-0-1 in seinem Bereich zu verwenden, einmal Heparin 10.000 I.E./24h i.v..

Fallvignette 8: *Pneumogene Sepsis und akute HIT-II, Thrombozytenabfall, Thrombosen, HIT-Schnelltest positiv.*

21 Freitextantworten: Am häufigsten wurde Argatroban (18-mal) genannt. Dabei gab ein Teilnehmer an, Argatroban „nicht PTT-wirksam“ zu verwenden und vier antworteten „nach PTT“, ohne ein Ziel zu benennen. Einmal wurde ein PTT-Zielwert von 25-40s angegeben, 1 x 45s, 1 x 50s, 3 x 60s, 1x 50-60s, 1 x 50-70s und 3 x 60-80s. Fondaparinux wurde in diesem Fall 2 x in der Dosierung von 7,5mg/d und 3 x in der Dosierung 2,5mg/d genannt. Auch Dabigatran (2x), Danaparoid (2x) und DOAKs (1x) kamen in den Freitextantworten vor.

Fallvignette 9: *Abdominelle Sepsis bei sekundärer Peritonitis, am 2. postoperativen Tag nach Fokussanierung, HIT-II in der Anamnese, Jahre zurückliegend, aktuell kein Hinweis auf thromboembolische Ereignisse oder Thrombozytenabfall.*

Insgesamt 26 Antworten. Sechs Antwortende gaben an, Argatroban mit dem Ziel zu verwenden, entweder „keine“ oder eine PTT von ≤ 40s zu erreichen. In einer teilnehmenden Klinik liegt der Zielwert bei <45s, in einer bei 40-60s und in zwei Kliniken bei 50-60s. Darüber hinaus wurden Enoxaparin in prophylaktischer Dosis (3x), einmal

Danaparoid (2 x 750IE s.c.), Fondaparinux (9x) in prophylaktischer Dosis und einmal Apixaban (2,5mg p.o. 1-0-1). Interessanterweise wurde von zwei Kliniken auch UFH (300 I.E./h bzw. 500 bis 800 iE/h) genannt. Eine Klinik gab an, nach der initialen Behandlung mit Argatroban, auf Danaparoid oder in ausgewählten Fällen im off-label use auf Fondaparinux umzustellen.

Fallvignette 10: *Abdominelle Sepsis bei sekundärer Peritonitis, am 2. postoperativen Tag nach Fokussanierung, akute HIT-II, Thrombozytenabfall, Thrombosen, HIT-Schnelltest positiv.*

Insgesamt wurden 27 Freitextantworten abgegeben. Dabei wurde 21-mal Argatroban genannt. Hierbei variierten die Zielparameter der Therapie (soweit sie angegeben wurden; 14x) signifikant. Während zwei der teilnehmenden Intensivbereiche Argatroban mit einem PTT-Ziel von <40s verabreichen, geben zwei Zielwerte von <45s bzw. 40-50s an. Zweimal wurde der PTT-Zielbereich von 40-60s, dreimal der von 50-60s und zweimal genau 60s genannt. In zwei Intensivbereichen werden sogar Zielbereiche von 60-70s angestrebt. In 4 Freitextantworten wurde Fondaparinux genannt. Zwei dieser Intensivbereiche nutzen dabei die prophylaktische Dosierung (1x2,5mg/d) und zwei die therapeutische (1x7,5mg/d). In einer Freitextantwort wurde Heparin (500 bis 800 IE/h) mit einer Ziel-PTT von 35s genannt.

ESM 7 Antikoagulation auf internistisch vs. anästhesiologisch geführten ITS bei vorbestehender Antikoagulation mit DOAK bei Vorhofflimmern.

„Keine Antikoagulation", „Danaparoid" sowie „Apixaban/Edoxaban od. Rivaroxaban" standen als weitere Antwortmöglichkeiten zur Verfügung, wurden aber von keinem Teilnehmer genannt. Aus Gründen der Übersichtlichkeit werden sie hier nicht dargestellt. DOAK = direkte orale Antikoagulanzien, IPK = Intermittierende pneumatische Kompression, MTPS = Medizinische Thromboseprophylaxestrümpfe, NMH = niedermolekulares Heparin, PTT = partielle Thromboplastinzeit, UFH = unfraktioniertes Heparin.

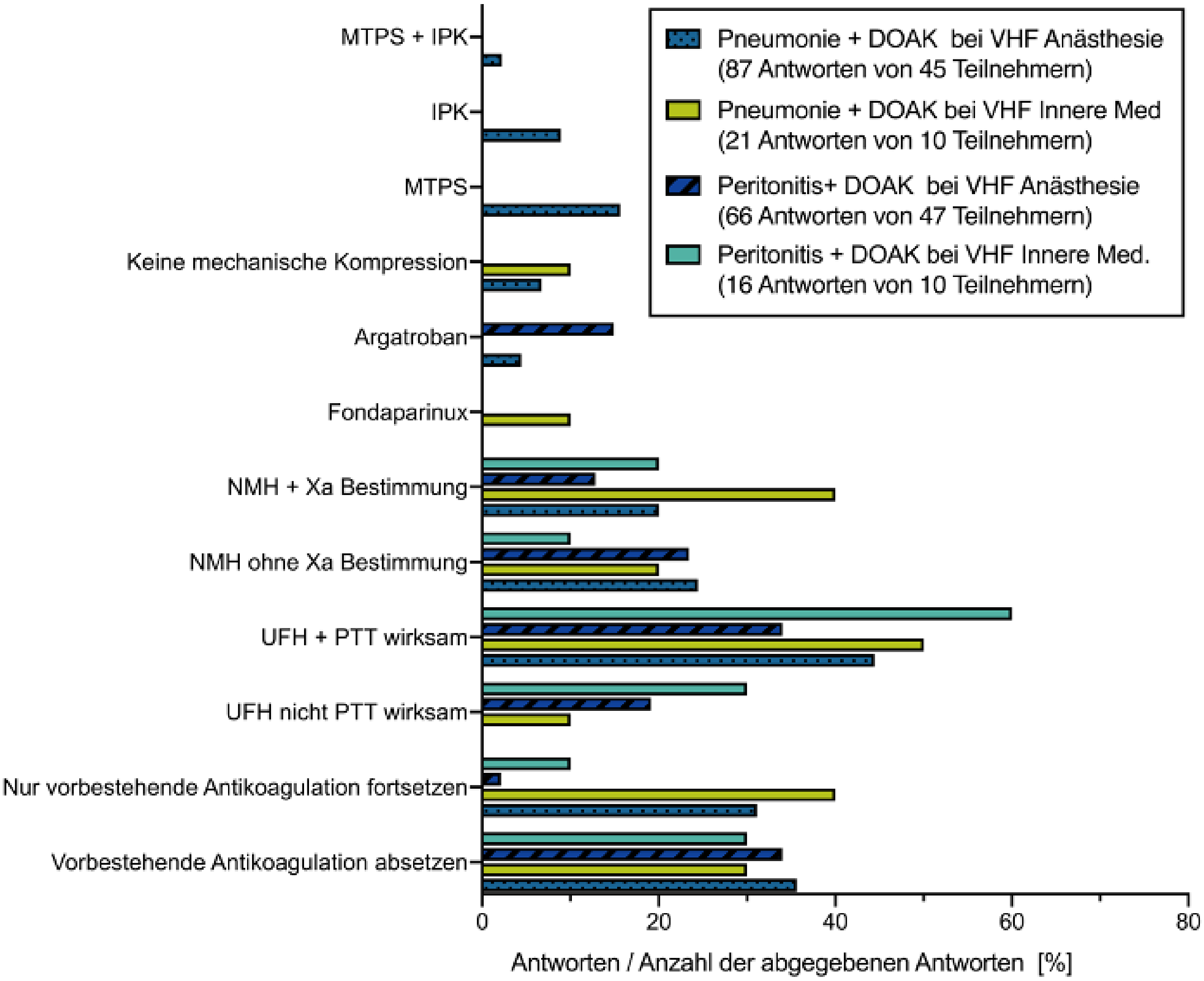

ESM 8: Antikoagulation auf internistisch vs. anästhesiologisch geführten ITS bei vorbestehender Antikoagulation mit Marcumar bei VHF

„Keine Antikoagulation", „Danaparoid" sowie „Apixaban/Edoxaban od. Rivaroxaban" standen als weitere Antwortmöglichkeiten zur Verfügung, wurden aber von keinem Teilnehmer genannt. Aus Gründen der Übersichtlichkeit werden sie hier nicht dargestellt. DOAK = direkte orale Antikoagulanzien, IPK = Intermittierende pneumatische Kompression, MTPS = Medizinische Thromboseprophylaxestrümpfe, NMH = niedermolekulares Heparin, PTT = partielle Thromboplastinzeit, UFH = unfraktioniertes Heparin.

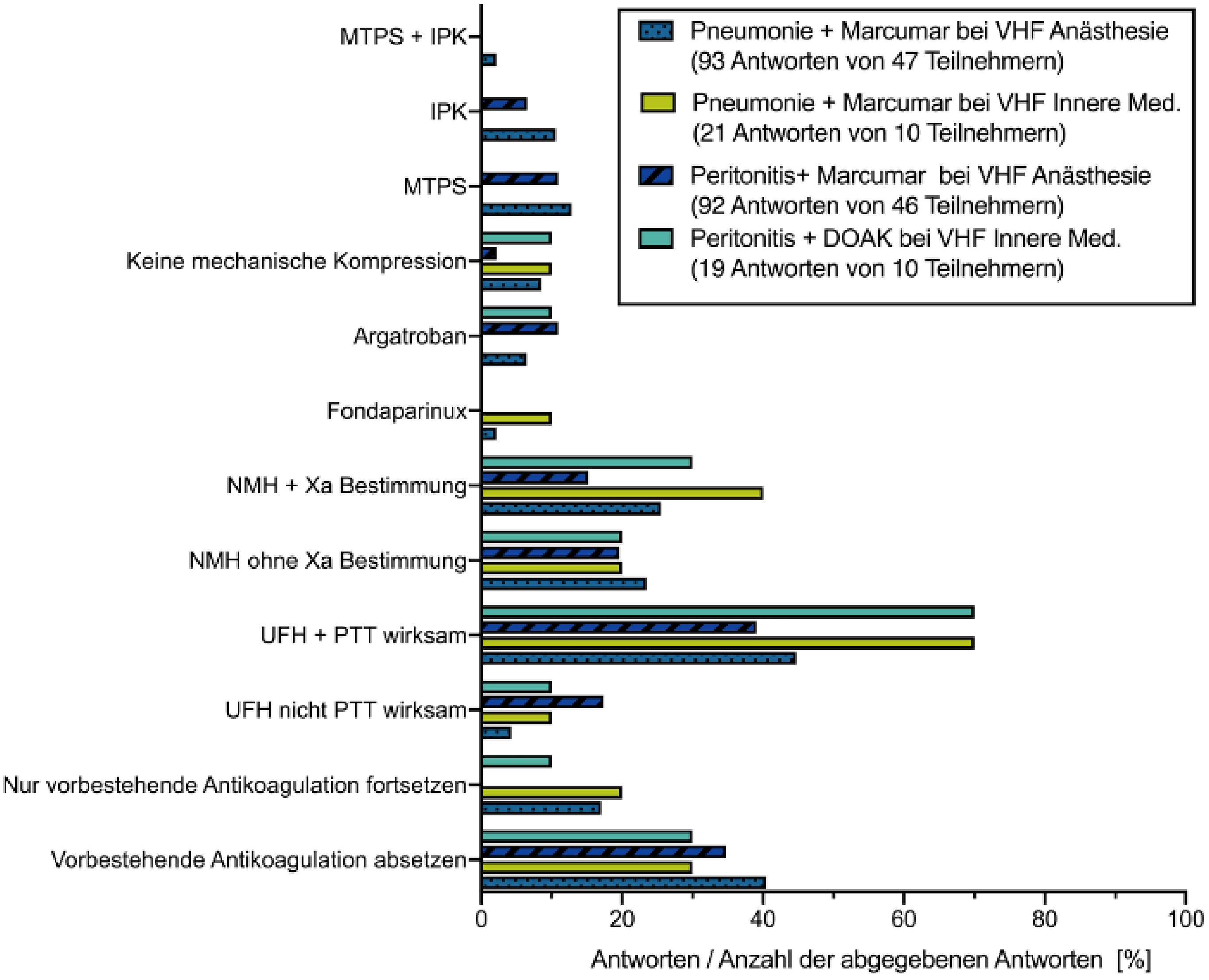

ESM9 Antikoagulation auf internistisch vs. anästhesiologisch geführten ITS bei akuter Heparin-induzierter Thrombozytopenie Typ 2

DOAK = direkte orale Antikoagulanzien, IPK = Intermittierende pneumatische Kompression, MTPS = Medizinische Thromboseprophylaxestrümpfe, NMH = niedermolekulares Heparin, PTT = partielle Thromboplastinzeit, UFH = unfraktioniertes Heparin.

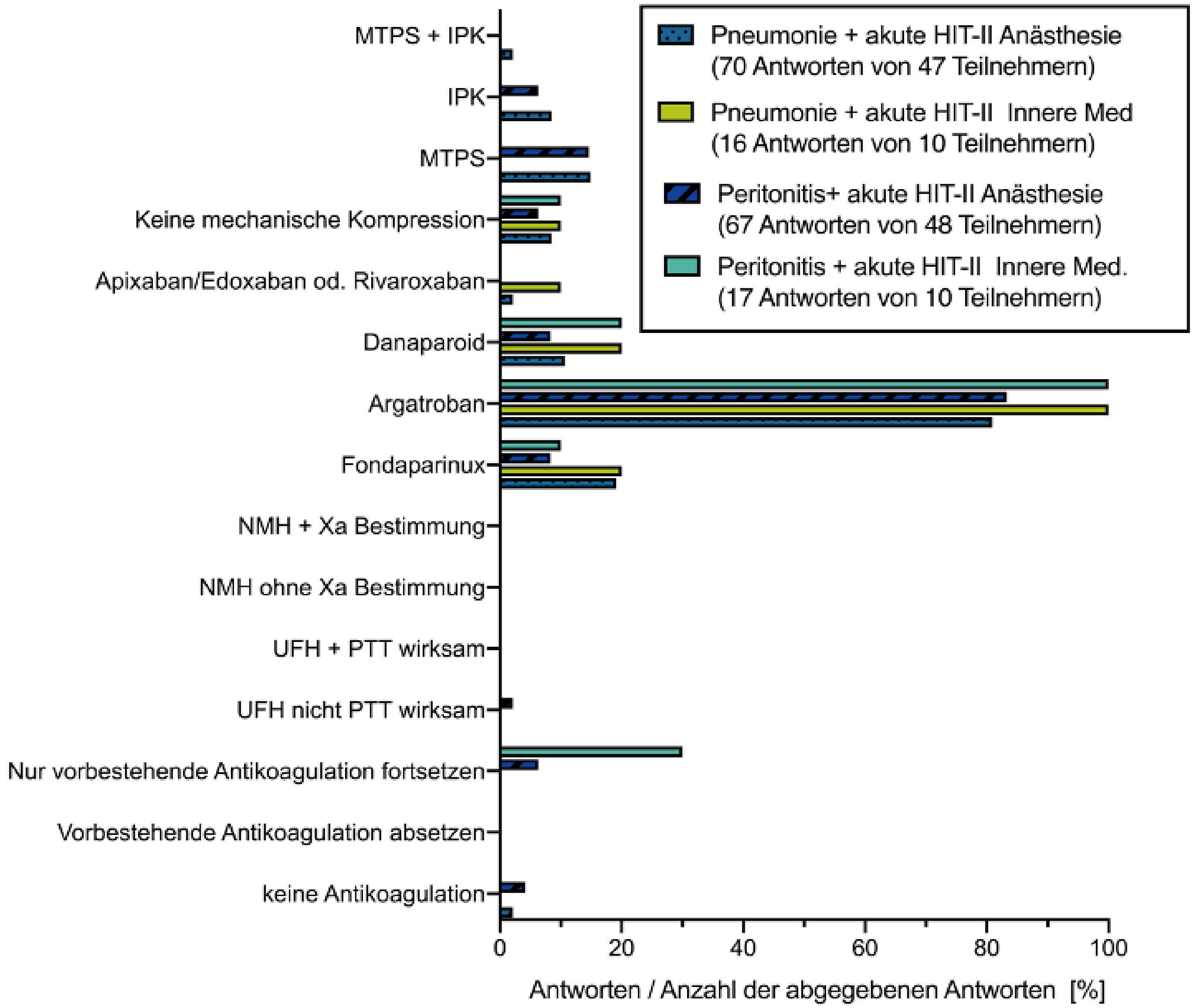

Supplement: Supplementary file 1 [file 101_2021_1011_MOESM1_ESM.pdf]
